# Aggregation-and-Attention Network for brain tumor segmentation

**DOI:** 10.1186/s12880-021-00639-8

**Published:** 2021-07-09

**Authors:** Chih-Wei Lin, Yu Hong, Jinfu Liu

**Affiliations:** 1 College of Computer and Information Science, Fujian Agriculture and Forestry University, Fuzhou, China; 2 College of Forestry, Fujian Agriculture and Forestry University, Fuzhou, China; 3 Forestry Post-Doctoral Station of Fujian Agriculture and Forestry University, Fuzhou, China; 4 Key Laboratory for Ecology and Resource Statistics of Fujian Province, Fuzhou, China

**Keywords:** Brain glioma, Image segmentation, Medical diagnosis, Convolution neural network

## Abstract

**Background:**

Glioma is a malignant brain tumor; its location is complex and is difficult to remove surgically. To diagnosis the brain tumor, doctors can precisely diagnose and localize the disease using medical images. However, the computer-assisted diagnosis for the brain tumor diagnosis is still the problem because the rough segmentation of the brain tumor makes the internal grade of the tumor incorrect.

**Methods:**

In this paper, we proposed an Aggregation-and-Attention Network for brain tumor segmentation. The proposed network takes the U-Net as the backbone, aggregates multi-scale semantic information, and focuses on crucial information to perform brain tumor segmentation. To this end, we proposed an enhanced down-sampling module and Up-Sampling Layer to compensate for the information loss. The multi-scale connection module is to construct the multi-receptive semantic fusion between encoder and decoder. Furthermore, we designed a dual-attention fusion module that can extract and enhance the spatial relationship of magnetic resonance imaging and applied the strategy of deep supervision in different parts of the proposed network.

**Results:**

Experimental results show that the performance of the proposed framework is the best on the BraTS2020 dataset, compared with the-state-of-art networks. The performance of the proposed framework surpasses all the comparison networks, and its average accuracies of the four indexes are 0.860, 0.885, 0.932, and 1.2325, respectively.

**Conclusions:**

The framework and modules of the proposed framework are scientific and practical, which can extract and aggregate useful semantic information and enhance the ability of glioma segmentation.

## Background

The brain is an essential organ in humans, responsible for controlling and coordinating body metabolism and activity, and also plays a function in cognition, thinking, and learning [1]. Glioma has emerged as one of the most major brain diseases that impair human health. It is closely related to the abnormal organization seen in the human brain [2, 3]. Modern medicine can help doctors judge the type and severity of brain tumors by acquiring information about brain tissue in non-invasive ways, such as medical imaging technology [4]. For example, magnetic resonance imaging (MRI) has high contrast in soft tissue imaging, such as nerve, blood vessel, and muscles, compared with other imaging techniques and can provide brain images with various modalities from the same patient [5]. Therefore, the study on image segmentation of brain tumors mainly focused on MRI [6, 7].

The requirement for rapid and accurate identification of diseases by computer technology is increasing due to the complexity of brain lesions [8]. Therefore, image segmentation is critical research in the field of computer vision. It refers to dividing an image into several non-overlapping subareas according to the pixel features, which satisfies the image discrimination requirements of glioma. The traditional methods of brain MRI segmentation mainly include threshold segmentation [9, 10], region segmentation [11, 12], and clustering analysis [13]. The common feature of these methods mainly relies on prior knowledge and low-level semantics to achieve simple brain segmentation tasks. However, the traditional segmentation methods cannot satisfy high accuracy requirements due to increased MRI resolution and content complexity.

In recent years, deep learning technology has gradually matured, leading to the emergence of models and algorithms for brain tumor segmentation based on a convolutional neural network (CNN) [14]. Unlike traditional segmentation methods, CNN does not require prior knowledge and can automatically extract and learn glioma features from different MRI modalities. U-Net [15] is the most common and effective basic framework with encoding–decoding structure, with uses skip connection to achieve the transmission of features between encoding and decoding. The current network of related medical image segmentation is improved based on U-Net. One is to improve the structure within encoding or decoding; for example, Res-Unet [16] increases the depth of the model by adding the skip connections in sampling modules. MultiRes U-Net [17] proposed a MultiRes Block, referring to Inception, to replace the basic modules. Others are optimizing the skip-connection between encoder and decoder; for example, U-Net++ [18] replaced the original long connections using short, dense connections similar to DenseNet [19], reducing semantic inconsistencies, U-Net 3+ [20] introduced full-scale skip-connections and made full use of multi-scale information in the encoder-decoder. In addition, there are some new sub-decoder routes to improve the network segmentation effect. Jiarui [21] studied the Variational Autoencoder (VAE) [22] and a two-stage cascaded U-net [23] structure to propose an end-to-end improved 3D-UNet.

**Fig. 1:**
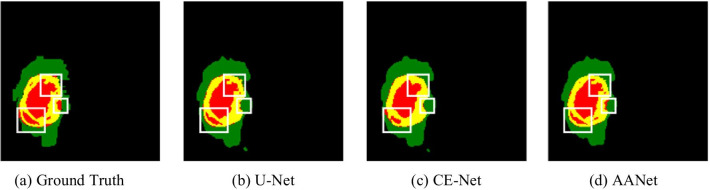
The visualization of ground truth and segmentation results with various methods. **a** The ground truth of brain tumor in three subareas, **b**–**c** segmentation results of U-Net and CE-Net. **d** Segmentation result of the proposed network. The white boxes mark the highlighted area, where shows that existing networks cannot accurately segment the grade and contour of brain tumor compared to AANet

These networks provide good segmentation results, but they are still inadequate for the segmentation tasks of brain tumors due to the process of network convolution often ignores the relationship between different modalities. Therefore, it is necessary to extract the feature differences between glioma and normal tissue and distinguish the differences between different grades of tumors within glioma. Furthermore, to separate precise tissue contours, the network requires extracting multi-scale semantic information as much as possible while reducing information loss during the convolution process. As shown in Fig. 1, existing networks, such as U-Net and CE-Net, cannot accurately segment the grade and contour of the brain. In order to solve these problems, we propose a novel network named Aggregation-and-Attention Network (AANet), which makes full use of features to improve segmentation performance. Its main contributions are as follows:

- We proposed an Aggregation-and-Attention Network (AANet), including the enhanced down-sampling (EDS) module, the multi-scale connection (MSC) module, and the dual-attention fusion (DAF) module.
- The EDS module decreases the lost information by skip-connection and fuses information for different convolutions in the same sampling layer.
- The MSC module extracts the context semantic information by considering the multi-receptive field, and that is sent to the downsampling to strengthen the semantic context. It is used to replace the skip connection.
- The DAF module is added to the network's bottom to increase the spatial and channel information through segmentation.
- We demonstrate state-of-the-art performances of the proposed AANet on BraTS2020. It shows that AAUnet could effectively extract information from brain MRI and segment tumors of different grades.

## Method

### Aggregation-and-Attention Network

**Fig. 2:**
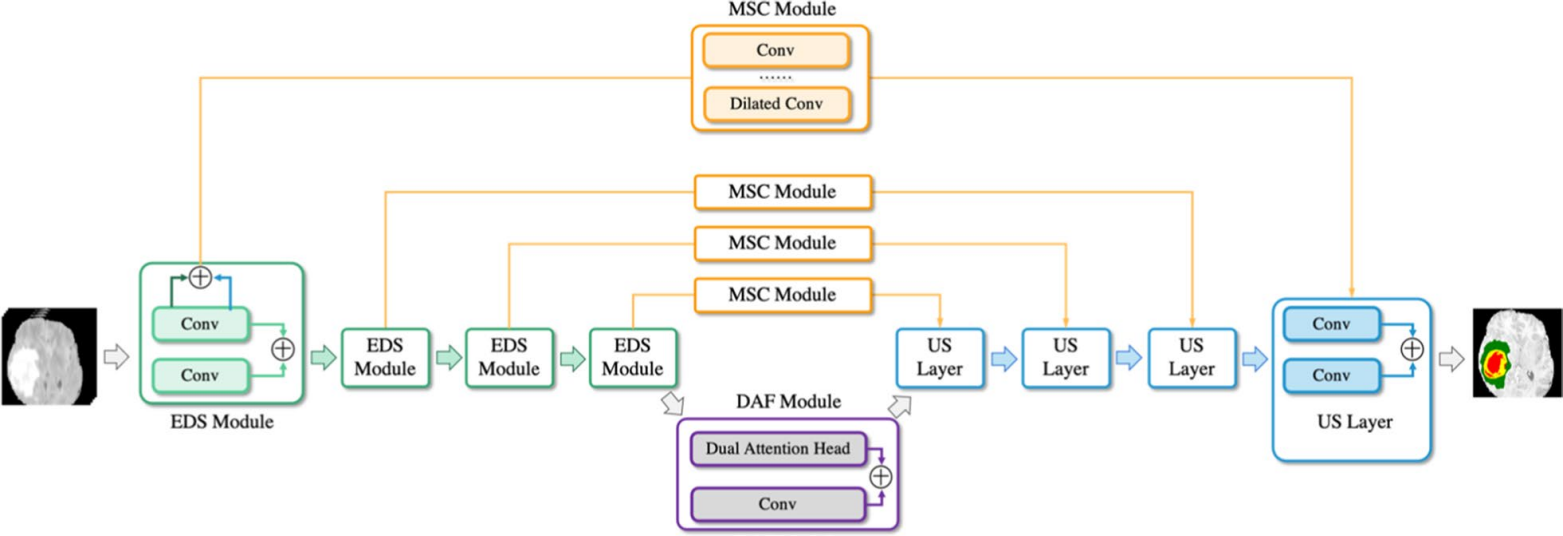
Architecture of the proposed AANet

The framework of the proposed Aggregation-and-Attention Network (AANet) for brain tumor segmentation is shown in Fig. 2. The network designed three main parts based on U-Net: enhanced down-sampling (EDS) module, multi-scale connection (MSC) module, and dual-attention fusion (DAF) module. First, the EDS module is constructed in the encoder, which fuses features of different locations within the same module to reduce information loss and improves encoding quality with deep supervision. Second, the DAF module is added at the bottom of encoding and decoding to highlight the critical feature information for location, channel, and fusion. Moreover, we replace the skip connection with the MSC module to transmit richer context semantic information. The decoding process is similar to encoding but only adds residual connection and deep supervision. These modules significantly improve the segmentation capability of the network. The details of the proposed module structures will be described in the following subsections.

### Enhanced down-sampling (EDS) module

The encoding process of U-Net plays an important role. The output of each down-sampling layer serves as the information basis for subsequent convolution and is also one of the input sources for the up-sampling layer in decoding. The network gradually extracts abstract high-level semantic information from rough low-level semantic information by adding more convolution and pooling operations but still has the problem of information loss. Therefore, we proposed the EDS module, which has two aspects: (1) compensating for information loss and (2) controlling encoding quality, to overcome these issues.

**Fig. 3:**
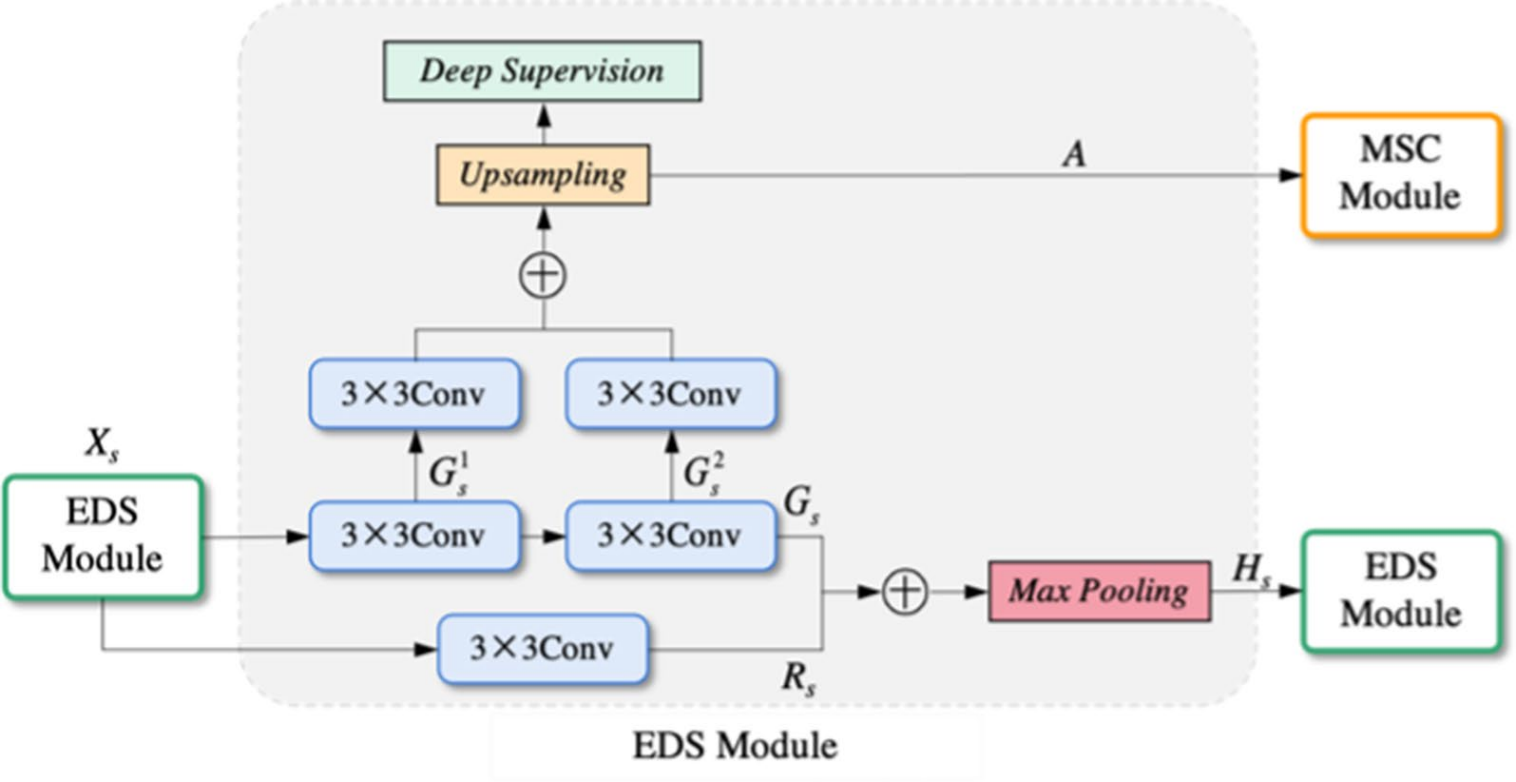
The architecture of the EDS module

The architecture of the EDS module is presented in Fig. 3. In Fig. 3, *X*_*s*_ $\in$ *R*^C×H×W^ is the input with the spatial size *s,* and *H*_*s*_ $\in$  *R*^C/2×H/2×W/2^ is the output and be presented as:

1 $$H_{s} = M\left( {G_{s} + R_{s} } \right) = M(l(\sigma (F(w_{s}^{2} ,l(\sigma (F(w_{s}^{1} ,X_{s} )))))) + l(\sigma (F(w_{s}^{3} ,X_{s} ))) + \beta _{s} )$$

where *G*_*s*_ and *R*_*s*_ are the feature maps during convolution, *F*(·,·) indicates convolution operation, *σ* denotes batch normalization, *w* is the convolution weight, *β* is the convolution bias, and *l* and* M* presents ReLU activation and max pooling, respectively. The Eq. (1) is also applied in the US Layer to fuse features in the encoder.

$G_{s}^{1}$ is the low-level feature and $G_{s}^{2}$ is the higher-level feature within *G*_*s*_ in which the previous studies usually ignored the differences between $G_{s}^{1}$ and $G_{s}^{2}$. For brain tumor segmentation, low-level semantics can optimize the details within the tumor, while high-level semantics can help segment the tumor's global area and contour. Therefore, the $G_{s}^{1}$ and $G_{s}^{{\text{2}}}$ are fused as:

2 $$A = U(l(\sigma (F(v_{s}^{1} ,G_{s}^{1} ))) \oplus l(\sigma (F(v_{s}^{2} ,G_{s}^{2} ))) + g_{s} )$$

where *A* $\in$ *R*^*C*×*H*×*W*^ is the fusion feature, *v* is the convolution weight,* g* indicates the bias of convolution operation, $\oplus$ presents feature concatenating, and *U* is the upsampling operation. In addition, we design the deep supervision for *A* to achieve the goal of controlling feature quality.

### Multi-scale connection (MSC) module

The skip connections between the same scale of encoding and decoding are the structural component of U-Net, where features from encoding are incorporated into the decoding process. The purpose is to merge the detail features extracted from encoding into decoding and restore the advanced semantic information through decoding operations. However, directly extracting the output features from encoding for simple addition cannot control the quality of the features, causing invalid noise features to spread in the network. Moreover, the context semantic information contained in the different levels of features is not fully explored.

**Fig. 4:**
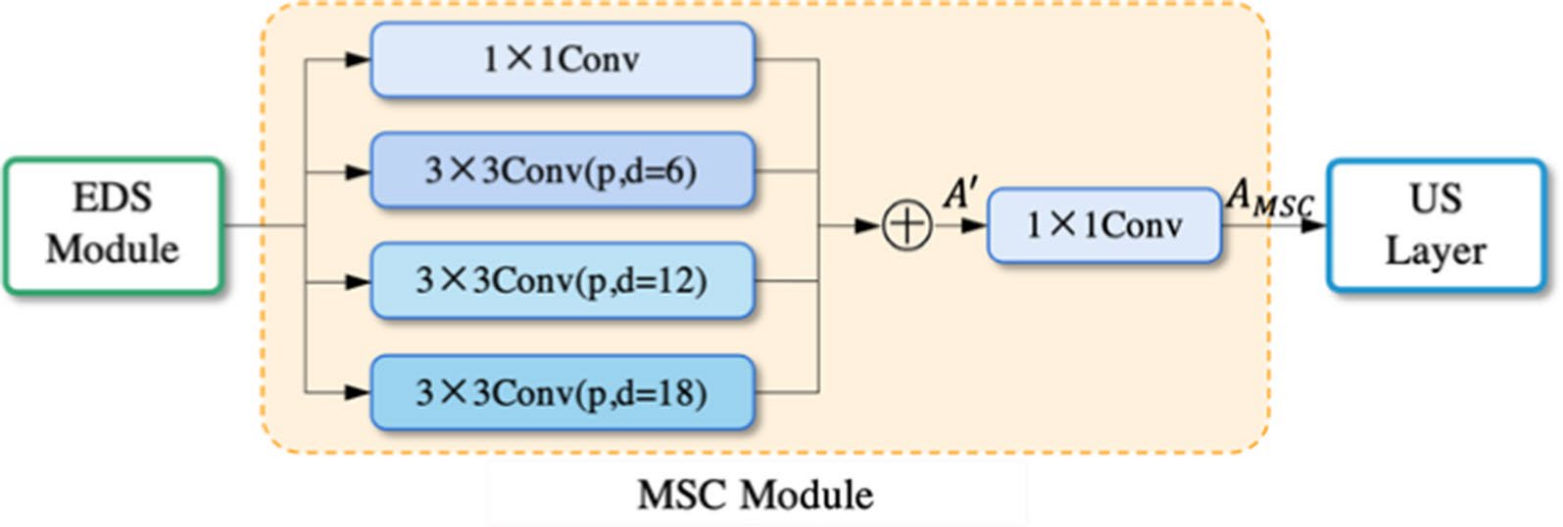
The architecture of the MSC module

In order to deliver high-quality features in the form of skip connections, we take $A \in R$^*C*×*H*×*W*^ from the EDS module as input, and the output *A*_*MSC*_ $\in$ *R*^*C*×*H*×*W*^ will send to the corresponding up-sampling layers, the calculating process can be formulated as:

3 $$A^{\prime} = A_{{1 \times 1}} \oplus A_{{3 \times 3}}^{{p,d = 6}} \oplus A_{{3 \times 3}}^{{p,d = 12}} \oplus A_{{3 \times 3}}^{{p,d = 18}}$$

4 $$A_{{MSC}} = l\left(\sigma \left( {F\left( {m,A^{\prime}} \right)} \right)\right) + \alpha$$

where *A*ʹ indicates the fusion feature, which concatenates *A*_*k*×*k*_, *A*_*k*×*k*_ is *A* through *k* × *k* convolution layers, *p* and *d* denote the size of padding and dilated rate, $\oplus$ presents feature concatenating, m is convolution weight, and *α* is convolution bias. The architecture of the MSC module is presented in Fig. 4.

### Dual-attention fusion (DAF) module

**Fig. 5:**
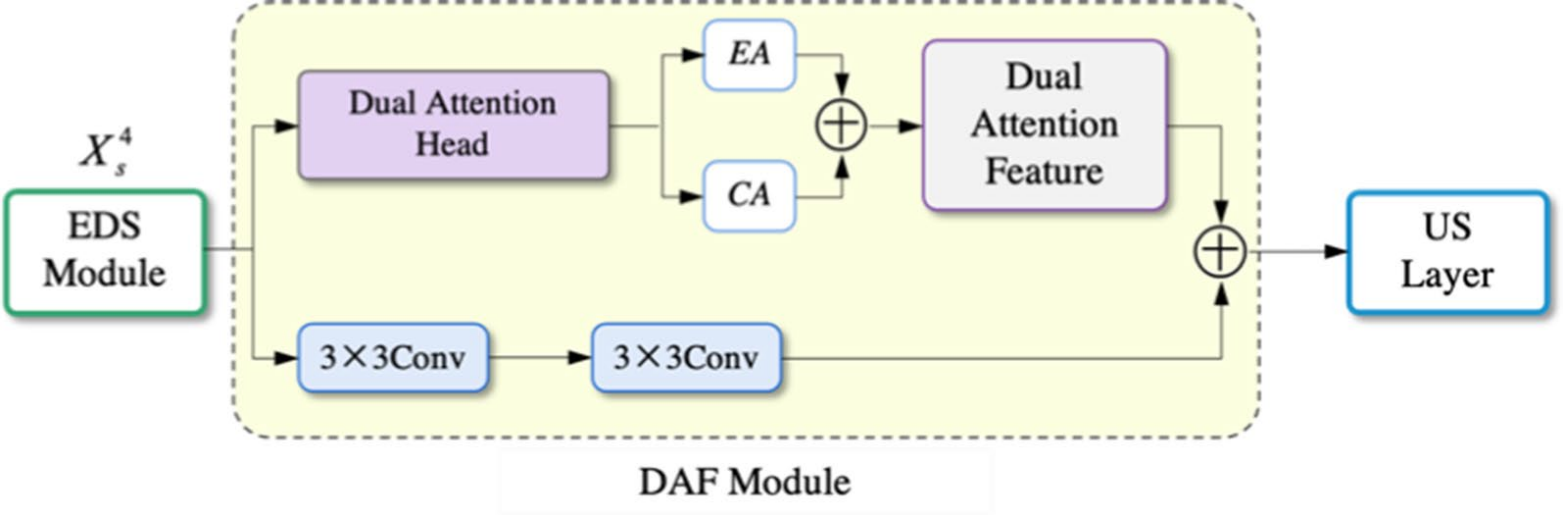
The architecture of the DAF module

The U-Net increases the number of convolution kernels to 1024 at the bottom connection to increase high-level information. However, the 3 × 3 convolution operation extracts features through a limited field of view without considering the correlation between the feature locations and channels. Therefore, we propose a Dual-Attention fusion (DAF) module, which applies two 3 × 3 convolutions for high-level semantic information, together with dual-attention heads to acquire positionally and channel attention features, respectively. The structure is shown in Fig. 5.

**Fig. 6:**
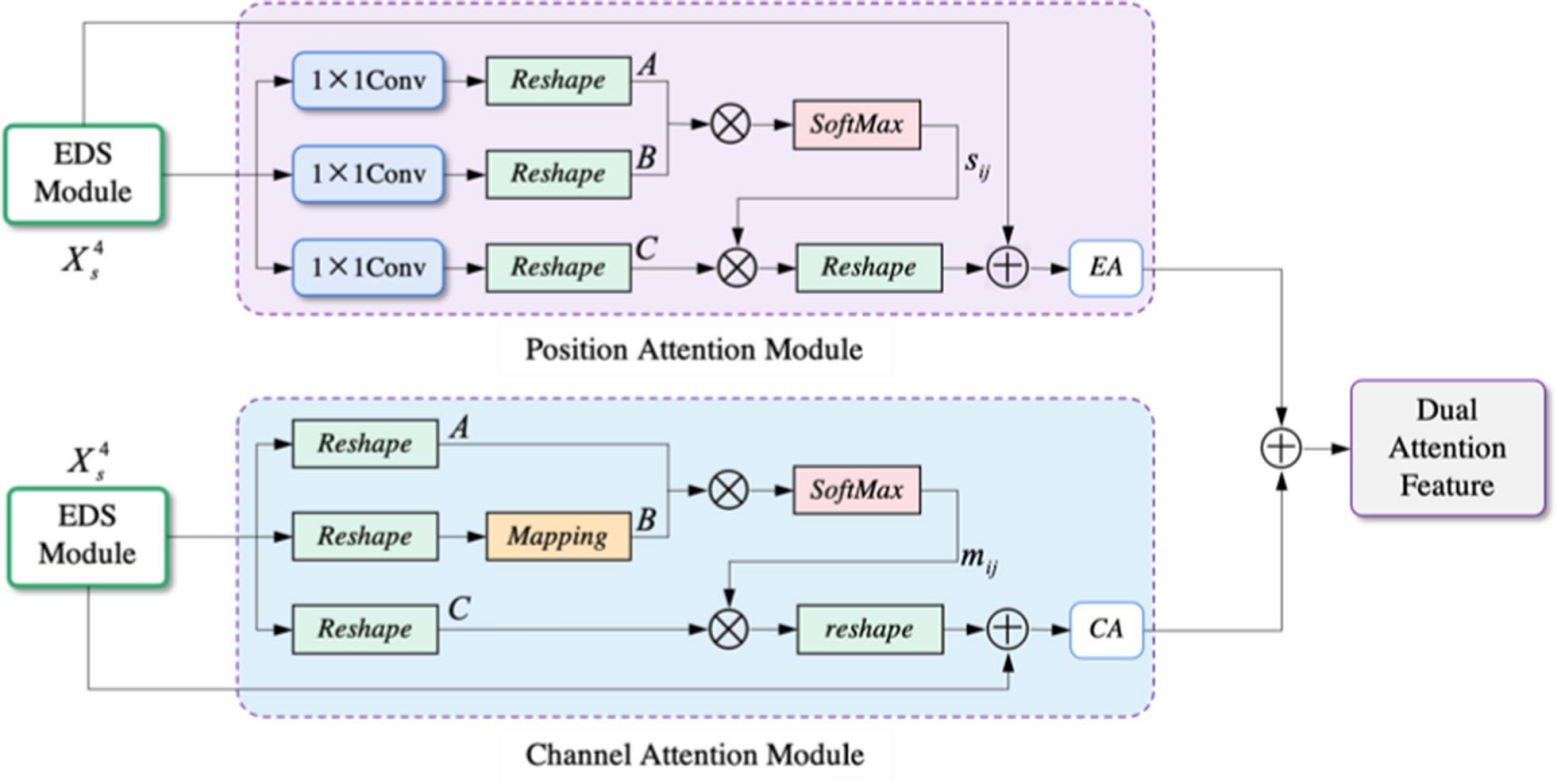
The architecture of the dual-attention head

The dual attention head includes a positional attention module and a channel attention module, as presented in Fig. 6. The positional attention (PA) module is on the upper half of Fig. 6. In Fig. 6, we toked $X_{s}^{4} \in R$^C×H×W^ as input and obtained the output *EA* $\in$  *R*^C×H×W^ from the PA module. This process of PA is summarized as:

5 $$S \in R^{{H \times W \times H \times W}} :~s_{{ji}} = \frac{{\exp \left( {A_{i} \cdot B_{j} } \right)}}{{\mathop \sum \nolimits_{{i = 1}}^{N} \exp \left( {A_{i} \cdot B_{j} } \right)}}$$

6 $$EA \in R^{{C \times H \times W}} :~EA_{j} = \alpha \mathop \sum \limits_{{i = 1}}^{N} \left( {s_{{ji}} C_{i} } \right) + (X_{s}^{4} )_{j}$$

where $S\in R$^(H×W)×(H×W)^ is spatial attention map, *s*_*ji*_ is used to measure the correlation between position *i* and position *j*, and the larger the value of *s*_*ji*_ means the higher the correlation. *A*  $\in$ *R*^C×(H×W)^, $B\in R$^C×(H×W)^, and $C\in R$^C×(H×W)^ indicate the different metric through 1 × 1 convolution followed by reshaping from $X_{s}^{4}$. *EA* is the position attention feature; *EA*_*j*_ represents the weighted sum of original features with the feature correlation between position *j* and all positions, integrating contextual location information into each point. *α* denotes scale factor.

The channel attention module locates on the lower half of Fig. 6, which does not use convolution to maintain the relationship between channels. The channel Attention module generates the output $CA\in R$^*B*×*C*×*H*×*W*^ and its procedure is summarized as:

7 $$M \in R^{{C \times C}} :~m_{{ji}} = \frac{{\exp \left( {A_{i} \cdot B_{j} } \right)}}{{\mathop \sum \nolimits_{{i = 1}}^{C} \exp \left( {A_{i} \cdot B_{j} } \right)}}$$

8 $$CA \in R^{{C \times H \times W}} :~CA_{j} = \beta \mathop \sum \limits_{{i = 1}}^{C} \left( {m_{{ji}} C_{i} } \right) + (X_{s}^{4} )_{j}$$

where $M\in R$^*C*×*C*^ is channel attention map, *m*_*ji*_ is used to measure the correlation between channel *i* and channel *j*, and the larger the value of *m*_*ji*_ means the higher the correlation. $A\in R$^*B*×(*H*×*W*)×*C*^ and $C\in R$^*B*×*C*×(*H*×*W*)^ indicate the different metric reshape from $X_{s}^{4}$, while $B\in R$^*B*×(*H*×*W*)×*C*^ is reshaped followed by mapping from $X_{s}^{4}$. Representing the weighted sum of original features with the feature correlation between channel *j* and all channels, which integrates the semantic dependence between channels into the feature map. *β* denotes scale factor.

### Up-Sampling Layer

**Fig. 7:**
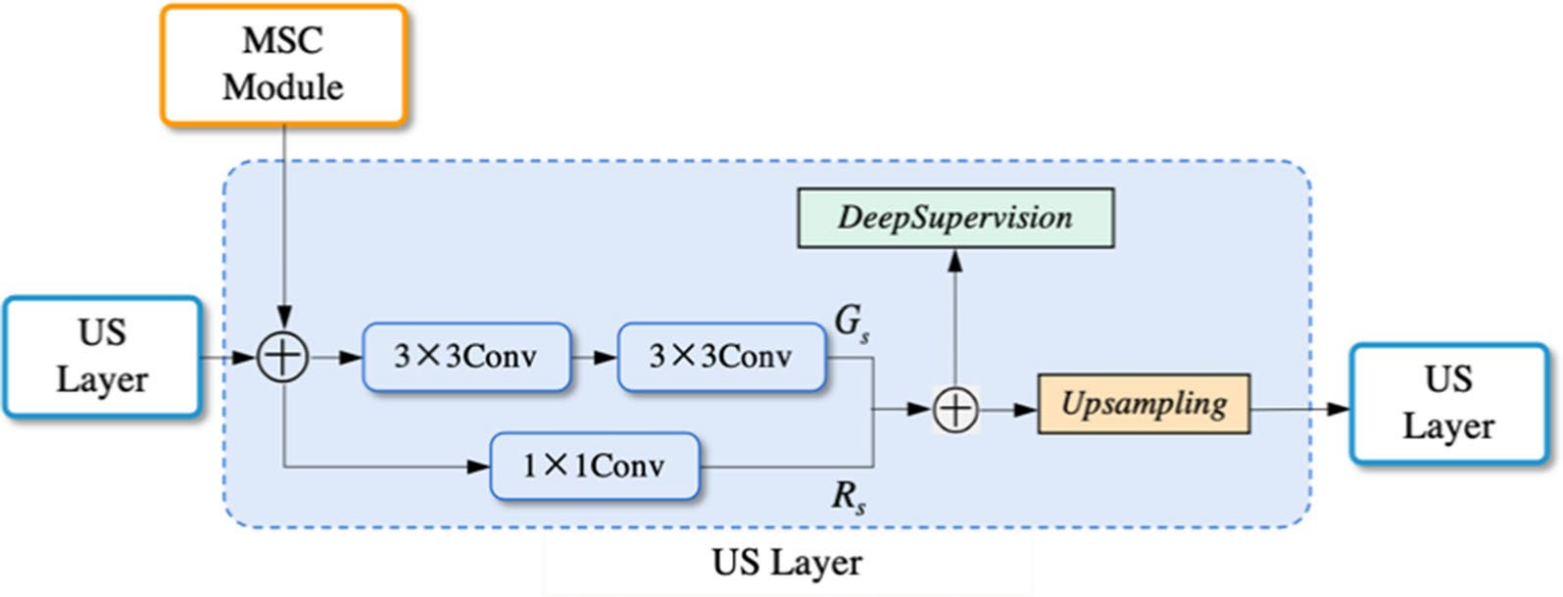
The architecture of the dual-attention head

Up-Sampling (US) Layer is similar to the EDS module's horizontal structure, described in “Enhanced down-sampling (EDS) module” section, and its structure is shown in Fig. 7. In Fig. 7, the residual connection is adopted in both US Layer and EDS modules, but the input sources are different. US Layers receive both feature maps from the previous US layer and MSC module at the same level. These feature maps firstly concatenate and executes Eq. (1). Mainly, deep supervision is also applied to control decoder quality.

### Loss function

This paper combines two kinds of loss functions to evaluate the segmentation effect: Binary Cross-Entropy (*L*_*BCE*_) and Dice loss (*L*_*Dice*_).

The equation of LBCE is defined as:

9 $$L_{{BCE}} = - \frac{1}{N}\mathop \sum \limits_{{i = 1}}^{N} y_{i} \cdot \log (p(y_{i} )) + \left( {1 - y_{i} } \right) \cdot \log (1 - p(y_{i} ))$$

where *y*_*i*_ denotes the prediction of pixel *i* (*i* = 1,…, *N*). If the prediction is consistent with the ground truth, then *y*_*i*_ = 1, otherwise *y*_*i*_ = 0. *p*(*y*_*i*_) is the probability when *y*_*i*_ = 1.

*L*_*Dice*_ can be calculated by:

10 $$L_{{D{\text{ice}}}} = \frac{{2 \times \sum T \cdot P}}{{\sum T^{2} + \sum P^{2} + \varepsilon }}$$

where *T* is the ground truth, *P* the prediction results, *ε* is the smoothing factor。

The final loss function used is designed as:

11 $$L = \alpha L_{{BCE}} + L_{{D{\text{ice}}}}$$

where *α* is constant and be set as 0.5.

We also apply *L* to evaluate the network when it carries out deep supervision. The total loss of model (*L*_*total*_) consists of four parts: the loss of the EDS module (*L*_*down*_), the loss of DAF module (*L*_*dual*_), the loss of US layers (*L*_*up*_), and the loss of final result (*L*_*result*_), and be formulated as:

12 $$L_{{total}} = \lambda _{1} \left( {L_{{down}} + L_{{up}} } \right) + \lambda _{2} L_{{dual}} + L_{{result}}$$

where *λ*_1_ and *λ*_2_ are constant, which are utilized to balance the contribution of each loss.

## Results

In this section, we first introduce the dataset and parameters of the proposed network. Then we compare the performance of the proposed network with several state-of-the-art networks to prove the efficiency of our network.

### Datasets and preprocessing

The BraTS2020 is an open dataset for brain tumor segmentation, which contains four modalities: the native (T1), T2-weighted (T2), the post-contrast T1-weighted (T1ce), and fluid-attenuated inversion recovery (FLAIR) images, [24–26]. In addition, there are three regions in one modality: the green area presents for the peritumoral edema (ED), the yellow area presents for the GD-enhancing tumor (ET), and the red area presents the Necrotic and Non-Enhancing Tumor (NCR/NET), as shown in Fig. 7.

The dataset contains three subsets, training, validation, and testing subsets. Training, validation, and testing subsets have 369, 125, and 166 MRIs with a size of 240 × 240 × 155, respectively. However, the validation and testing subset do not have corresponding ground truth. Therefore, we redivide the training data to achieve training and testing in different models. First, we normalized the original data to *N*(0,1) and then cropped with the center point as the original point to obtain the data blocks with a size of 155 × 160 × 160. Next, we slice data blocks along the Z-axis and generates 155 brain images of 160 × 160 for each sequence. Then, according to the order of slicing, we extract one slice from the four sequences respectively and combine the images into the size of 160 × 160 × 4.

### Implementation details

To ensure the comparability of experimental results, we set the training step as 100 with batch size 16 and use two GPU on both the proposed network and other networks. In order to prevent overfitting, we consider the strategy of the early stop and set the stop threshold as 20 based on the trend of validation accuracy. Moreover, we use Adam with an initial learning rate of 3e-4 as the network optimizer and set *λ*_1_ and *λ*_2_ as 0.4 and 0.2. For the training data of BraTS2020, we redivided the data set proportionally to obtain 17,519, 4,379, and 5,735 for training, validation, and testing. Notice that we use the same parameters setting and loss function as shown in Eq. 12 for all networks in the experiments.

### Evaluation

We take four indexes to evaluate the segmentation accuracy for a comprehensive and objective evaluation of the results: Dice Coefficient, Precision, Sensitivity, and Hausdorff Distance. These indexes are defined as follows:

13 $$Dice\;Coefficient = \frac{{2 \times TP}}{{2 \times TP + FP + FN}}$$

14 $$Precision = \;\frac{{TP}}{{TP + FP}}$$

15 $$Sensitivity = \frac{{TP}}{{TP + FN}}$$

16 $$Hausdorff\;Distance = d_{H} \left( {L,P} \right)$$

where *TP*, *FP,* and *FN* indicate true positive, false positive, and false negative. *d*_*H*_(·) denotes the operation of taking the minimum and the maximum. *L* and *P* present ground truths and predictions.

**Fig. 8:**
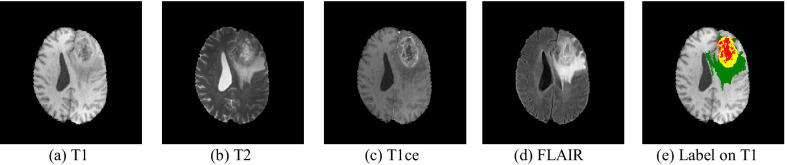
Visualization of one patient in four modalities in BraTS2020 training Dataset. (a) T1 MRI, (b) T2 MRI, (c) T1ce, (d) FLAIR MRI, and the label shown in T1 MRI

The main target of BraTS2020 is to consider the segmentation results of three-part: enhancing tumor (ET), core tumor (CT), and whole tumor (WT) in which ET, CT, WT represents as “red,” “red + yellow,” and “red + yellow + green” as shown in Fig. 8. Therefore, we evaluate the segmentation results of each part with these four indexes.

### Performance comparison

To demonstrate the performance of the proposed network, we compare several state-of-the-art networks with open-source code. We selected classic networks including FCN8s, U-Net, SegNet, andPSPNet; networks released in recent years like Refinenet, Deeplabv3, UNet2+, and DeepResUnet, the most advanced networks like CE-Net, CLCINet, and UNet3+, as the comparisons.

The comparison results are shown in Table 1. In Table 1, our network, AANet, achieves a remarkable performance on ET, CT, and WT with various indexes in which the AANet's precision and Hausdorff are 1.41% and 0.66 higher than U-Net on ET. Although our method is 0.08% lower than the best approach (RefineNet) on CT with the Sensitivity index, it is better than other networks' results on WT, CT, and ET with various indexes.

**Table 1:** Comparison on different networks with various indexes

Method	Year	Dice Coefficient↑			Precision↑			Sensitivity↑			Hausdorff Distance↓		
		WT	CT	ET	WT	CT	ET	WT	CT	ET	WT	CT	ET
FCN8s [27]	2015	0.5621	0.8317	0.4954	0.5644	0.8848	0.4896	0.9509	0.9037	0.9224	2.1830	1.1101	2.3330
UNet [15]	2015	0.7711	0.8386	0.6984	0.7806	0.9098	0.6767	0.9206	0.8799	0.8873	1.9039	1.2931	2.1166
SegNet [28]	2016	0.7655	0.8398	0.6907	0.7872	0.9260	0.6904	0.9279	0.8669	0.9066	1.3939	1.1268	1.9274
PSPNet [29]	2016	0.8177	0.8597	0.7394	0.8469	0.9394	0.7506	0.9013	0.8716	0.8629	1.6102	1.0735	1.7431
Refinenet [30]	2017	0.6974	0.8641	0.6371	0.7029	0.9165	0.6344	0.9472	**0.9127**	0.9212	1.7706	1.0306	1.9197
DeepLabV3 [31]	2017	0.6438	0.8484	0.5670	0.653	0.9166	0.5673	0.9250	0.8854	0.8852	2.2162	1.1124	2.3405
UNet 2 + [18]	2018	0.7881	0.8679	0.7253	0.8091	0.9323	0.7306	0.9369	0.8953	0.9168	1.4899	1.0451	1.6639
DeepResUNet [32]	2018	0.8061	0.8869	0.7510	0.8205	0.9456	0.7513	0.9452	0.9089	0.9289	1.4491	0.9693	1.5954
CE-Net [33]	2019	0.7299	0.857	0.6706	0.7423	0.9114	0.6725	0.9390	0.9038	0.9160	1.6851	1.0729	1.8375
CLCINet [10]	2019	0.7502	0.8562	0.6952	0.7585	0.917	0.6934	0.9514	0.9092	0.9334	1.6435	1.0452	1.7664
UNet 3 + [20]	2020	0.7992	0.8762	0.7412	0.8122	0.9379	0.7380	0.9472	0.9044	0.9322	1.4692	0.9968	1.6368
AANet	**-**	**0.8691**	**0.8956**	**0.8142**	**0.8855**	**0.9516**	**0.8177**	**0.9496**	0.9119	**0.9335**	**1.3078**	**0.9336**	**1.4561**

The best results are marked with bold

^**^↑ Indicates that the greater the index value, the better the network segmentation performance.↓ Indicates that the smaller the index value, the better

The network segmentation performance

**Fig. 9:**
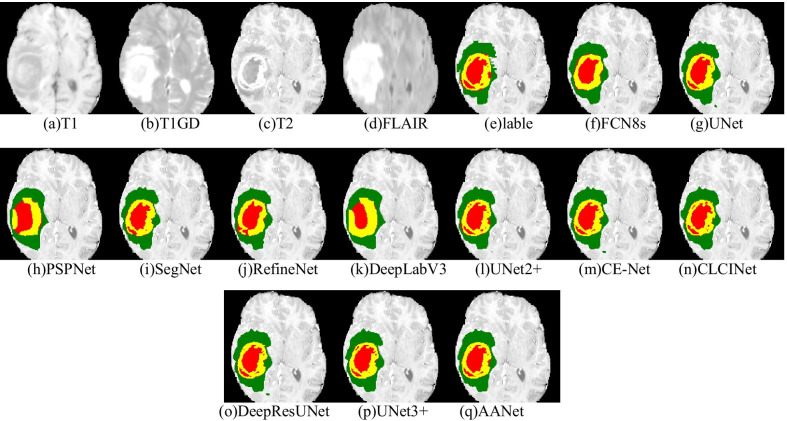
Visualization of segmentation results with different networks

Figure 9 shows the visualization results of the segmentation with different networks. FCN8s, PSPNet, and DeeplabV3 could only segment the general area of glioma, which differed significantly compared to the ground truth. SegNet, RefineNet, and CE-Net further refined the boundary contour of different tissues in glioma but did not segment the scattered edema area. On the other hand, UNet2+, DeepResUnet, CLCINet, and UNet3 + were very close to the ground truth, sensitive to discrete edema areas, and prone to segmentation confusion between different tumor regions. On this basis, AANet can restore the tortuous contour at the junction of different tumor regions and divide different tumor regions accurately and achieve a better overall segmentation effect. Therefore, AANet has a better MRI segmentation effect for a brain tumor compared with other networks.

## Discussion

We conduct the ablation experiments, including (1) effectiveness of basic modules, (2) ablation of MSC modules position, (3) comparison of DAF modules to verify the scientificity of each module.

### Effective of basic modules

We firstly take the U-Net as the baseline, which is our backbone, to demonstrate the effect of the proposed modules and present the results in Table 2. We add the proposed modules step by step with only *L*_*result*_ and then use deep supervisions with *L*_*down*_ and *L*_*up*_. In Table 2, the networks with the proposed modules achieve significant improvements compare to the baseline. EDS module plays a vital role in boosting the network's performance mostly, which is 3.9% higher than U-Net in the mean of Dice. It reflects that the EDS module can extract and transport useful information during training.

**Table 2:** The ablation experiments of basic modules

EDS	UPL	DAF	MSC	*L*_*down*_	*L*_*up*_	Dice↑			Precision↑			Sensitivity↑			Hausdorff↓		
						WT	CT	ET	WT	CT	ET	WT	CT	ET	WT	CT	ET
Baseline						0.771	0.839	0.698	0.781	0.910	0.677	0.921	0.880	0.887	1.904	1.293	2.117
+						0.800	0.883	0.741	0.814	0.937	0.743	0.946	0.913	0.926	1.488	0.981	1.650
+	+					0.808	0.880	0.751	0.828	0.935	0.760	0.943	0.913	0.922	1.423	0.986	1.569
+	+	+				0.818	0.874	0.758	0.836	0.932	0.760	0.944	0.902	0.926	1.401	1.033	1.574
+	+	+	+			0.829	0.868	0.770	0.841	0.924	0.770	0.946	0.904	0.925	1.397	1.064	1.560
+	+	+	+	+		0.849	0.891	**0.849**	0.860	0.945	0.792	**0.952**	**0.913**	**0.934**	1.360	**0.929**	1.507
+	+	+	+	+	+	**0.869**	**0.896**	0.814	**0.886**	**0.952**	**0.818**	0.950	0.912	**0.934**	**1.308**	0.934	**1.456**

The best results are marked with bold

^**^↑ indicates that the greater the index value, the better the network segmentation performance.↓ indicates that the smaller the index value, the better

The network segmentation performance. The *L*_*dual*_ in the DAF module is considered as an inherent attribute

Furthermore, the network with deep supervision achieves the best results in all indexes, demonstrating the benefits of controlling the feature quality. Networks with *L*_*down*_ and *L*_*up*_ achieve optimal in most indexes and suboptimal in other indexes. Therefore, we simultaneously consider these four modules with deep supervision in AANet as our framework.

### Ablation of MSC modules position

To understand the effect of the proposed MCS module, we consider taking various numbers of the MCS modules, setting them at different positions of the network, and demonstrating the analyzing results in Table 3. In Table 3, the network with four MSC modules achieves the best segmentation results with all indexes except for the Sensitivity index. However, the network's performance with four-position MSC modules is 0.4% lower than baseline in the WT and ET of Sensitivity and is 0.7% lower than the network with three-position MSC modules in CT of Sensitivity. The main reason is that the Sensitivity index focuses on presenting the model's ability to identify positive examples. Moreover, the semantic information captured by the MSC module is becoming increasingly scarce with the reduction of the size of the feature map. Furthermore, the dilated convolution may capture invalid information and thus affect the prediction of the true positive.

**Table 3:** The ablation experiments of MCS module’s position

1	2	3	4	Dice↑			Precision↑			Sensitivity↑			Hausdorff↓		
				WT	CT	ET	WT	CT	ET	WT	CT	ET	WT	CT	ET
Baseline				0.806	0.886	0.750	0.814	0.938	0.745	**0.954**	0.913	**0.938**	1.485	0.942	1.633
√				0.856	0.890	0.799	0.872	0.941	0.803	0.947	0.913	0.929	1.343	0.940	1.498
√	√			0.846	0.890	0.790	0.858	0.949	0.788	0.951	0.907	0.937	1.371	0.953	1.526
√	√	√		0.844	**0.896**	0.791	0.855	0.946	0.792	0.951	**0.919**	0.932	1.373	0.924	1.513
√	√	√	√	**0.869**	**0.896**	**0.814**	**0.886**	**0.952**	**0.818**	0.950	0.912	0.934	**1.308**	**0.934**	**1.456**

The best results are marked with bold

^**^1 ~ 4 are the positions of the MSC module. The smaller the feature map processed by the MSC module, the larger the corresponding position number value. For example, 1 represents the position connected with the first EDS module with the largest feature map

### Comparison of DAF modules

**Fig. 10:**
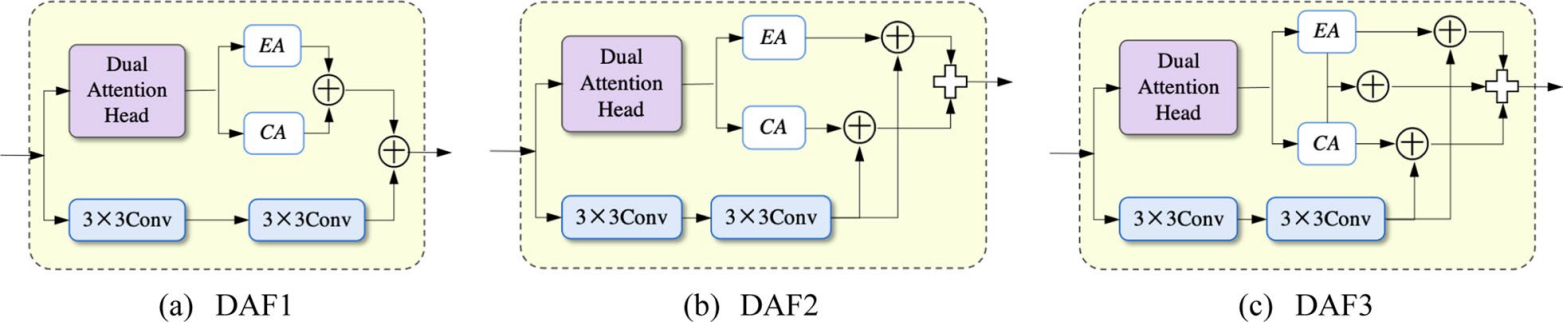
The architecture of DAFs

In this subsection, we analyze three variants of the DAF (DAF1, DAF2, and DAF3) module, demonstrate the frameworks of these variants in Fig. 10, and present the quantitative results in Table 4. In Fig. 10, DAF1 is the standard version used in AANet; DAF2 separately adds EA and CA with another feature map and then concatenates two parts; DAF3 absorbs the characteristics of DAF1 and DAF2. In Table 4, DAF1 outperforms the other two DAFs in most segmentation results. Although DAF3 also achieved the best accuracy in some regions of indexes, the calculation parameters are more outstanding than DAF1. Therefore, we adopt DAF1 as the underlying structure of AANet.

**Table 4:** The comparison of DAF modules’ variants

Module	Dice↑			Precision↑			Sensitivity↑			Hausdorff↓		
	WT	CT	ET	WT	CT	ET	WT	CT	ET	WT	CT	ET
DAF1	**0.869**	0.896	**0.814**	**0.886**	**0.952**	**0.818**	**0.950**	**0.912**	**0.934**	**1.308**	0.934	**1.456**
DAF2	0.853	0.891	0.796	0.870	0.949	0.799	0.947	0.910	0.930	1.346	0.944	1.493
DAF3	0.857	**0.898**	0.800	0.871	0.950	0.803	**0.950**	**0.912**	0.930	1.333	**0.919**	1.480

The best results are marked with bold

## Conclusion

This paper proposes an effective Aggregation-and-Attention Network (AANet) for Brain Tumor Segmentation based on U-Net. In order to solve the problems of unclear boundary and easy confusion of tumor division in the segmentation processing, we first proposed an enhanced down-sampling (EDS) module, which compensates for the loss of information and controls the coding quality. Moreover, we design the multi-scale connection (MSC) module to replace the skip-connection. The MSC module considers the multi-receptive field to extract the context semantic information, and that is sent to the downsampling to strengthen the semantic context. The dual attention fusion (DAF) module is designed to increase the attention information of positions and channels. Experimental results show that the performance of the proposed AANet is better than the most commonly used and advanced network frameworks on the BraTS2020 dataset.

To the best of our knowledge, there are existing intelligent recognition technologies to solve the problems of tumor cell recognition [34–36] but lost the intelligent segmentation technology to identify brain tumor cells existing in the brain edema area. Moreover, intelligent segmentation technology has been applied in the segmentation of COVID-19 infected areas of the lung on CT and X-ray images [37], similar to judging whether there are tumor cells in non-enhanced tumors and tumor edema areas. Therefore, we will attempt to collect the histopathological image of glioma in non-enhanced tumors and tumor edema areas to construct a glioma tumor cells dataset and verify the ability of AANET in cell segmentation to improve our segmentation algorithm in the future.

## Data Availability

The datasets analyzed during the current study are available in the BraTS2020 repository, https://www.cbica.upenn.edu/BraTS20/.

## Abbreviations

AANet: Aggregation-and-Attention Network
EDS module: Enhanced down-sampling module
US Layer: Up-Sampling Layer
MSC module: Multi-scale connection module
DAF module: Dual-attention fusion module
CNN: Convolutional neural network
VAE: Variational Autoencoder
PA: Positional attention
CA: Channel attention
ED: Peritumoral edema
ET: Enhancing tumor
NCR/NET: Necrotic and Non-Enhancing Tumor
CT: Core tumor
WT: Whole tumor
ET: Enhanced tumor
